# Supplementary material for: Proton-coupled transport mechanism of the efflux pump NorA
Source: Nat Commun. 2024 May 27;15:4494. doi: 10.1038/s41467-024-48759-3 (PMC11130294; doi:10.1038/s41467-024-48759-3)
Supplement: Supplementary file 1 — Supplementary Information [file 41467_2024_48759_MOESM1_ESM.pdf]

SUPPLEMENTARY INFORMATION

Proton-coupled transport mechanism of the efflux pump NorA

Jianping Li[1], Yan Li[2], Akiko Koide[3,4], Huihui Kuang[5], Victor J. Torres[6,7], Shohei Koide[3,8], Da-Neng Wang[2*], Nathaniel J. Traaseth[1*]

[1] *Department of Chemistry, New York University, New York, NY, USA*

[2] *Department of Cell Biology, New York University School of Medicine, New York, NY, USA*

[3] *Perlmutter Cancer Center, New York University School of Medicine, New York, NY, USA*

[4] *Department of Medicine, New York University School of Medicine, New York, NY, USA*

[5] *Simons Electron Microscopy Center, New York Structural Biology Center, New York, NY, USA*

[6] *Department of Microbiology, New York University School of Medicine, New York, NY, USA*

[7] *Antimicrobial-Resistant Pathogens Program, New York University School of Medicine, New York, NY, USA*

[8] *Department of Biochemistry and Molecular Pharmacology, New York University School of Medicine, New York, NY, USA*

* Corresponding authors: da-neng.wang@med.nyu.edu, traaseth@nyu.edu

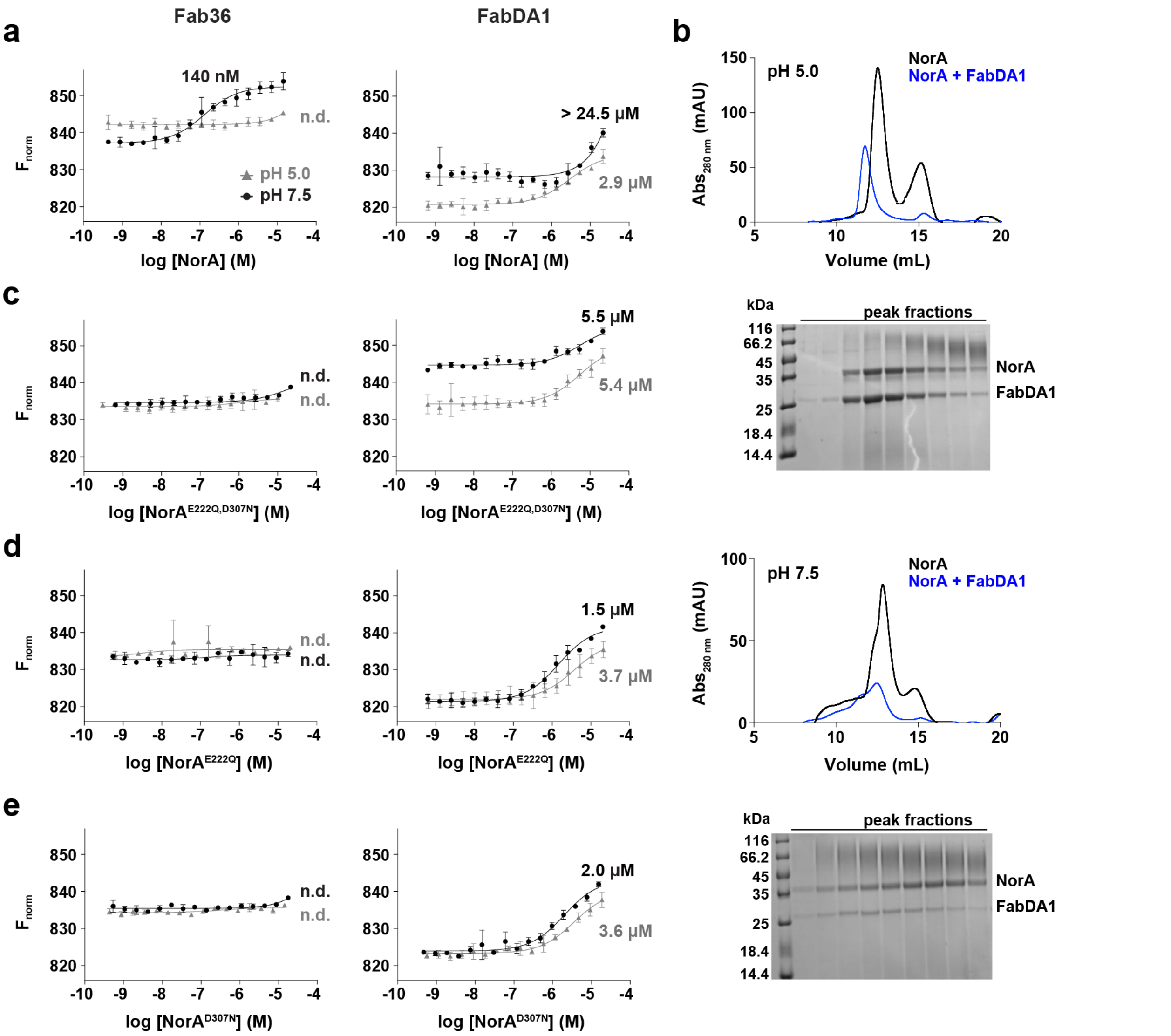

Supplementary Figure 1. Binding affinity measurement of NorA and mutants with Fab36 or FabDA1.

a, c-e. MST binding curves for fluorescently labeled Fab36 (left) and FabDA1 (right) binding to the indicated NorA variants, including NorA (a), $NorA^{E222Q,D307N}$ (c), $NorA^{E222Q}$ (d), and $NorA^{D307N}$ (e). NorA was reconstituted in PMAL-C8 amphipol at pH 5.0 (grey) or pH 7.5 (black). The K_d values are provided next to each dataset; 'n.d.' denotes cases where the K_d value could not be determined. The error range for K_d values, representing a 95% confidence interval of the non-linear fit, are as follows: 120 - 160 nM for NorA-Fab36 at pH 7.5, 1.9 - 4.6 μM for NorA-FabDA1

at pH 5.0, 2.8 - 11.2 μM for NorA^{E222Q,D307N}-FabDA1 at pH 5.0, 2.6 - 12.8 μM for NorA^{E222Q,D307N}-FabDA1 at pH 7.5, 2.0 - 7.2 μM for NorA^{E222Q}-FabDA1 at pH 5.0, 1.1 - 2.0 μM for NorA^{E222Q}-FabDA1 at pH 7.5, 2.4 - 5.4 μM for NorA^{D307N}-FabDA1 at pH 5.0, and 1.3 - 3.0 μM for NorA^{D307N}-FabDA1 at pH 7.5. The K_d value for NorA-FabDA1 at pH 7.5 could not be accurately fitted, and the K_d value was estimated to exceed the highest concentration of NorA in the experiment (> 24.5 μM). All MST binding experiments were conducted with three independent runs. NorA binding data to Fab36 at pH 7.5 in (a) was previously published[1].

b. SEC chromatograms depicting NorA reconstituted in PMAL-C8 amphipol alone (black traces) and NorA in the presence of three-fold molar excess of FabDA1 (blue traces) at two distinct pH conditions, pH 5.0 (top) and pH 7.5 (bottom). Additionally, Coomassie-stained SDS-PAGE gels display the peak fractions obtained from the corresponding SEC chromatogram of NorA and FabDA1 experiments.

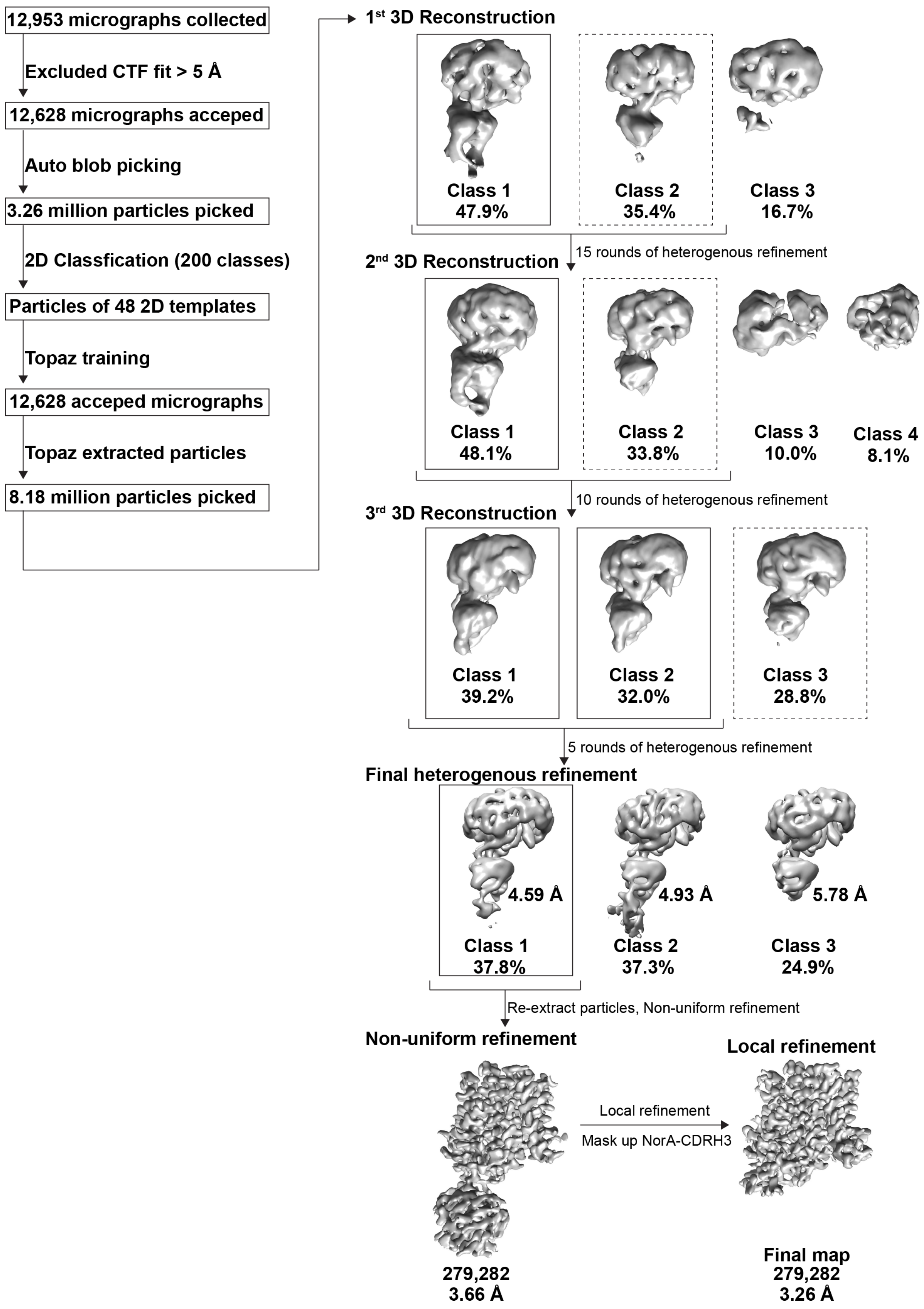
12,953 micrographs collected
Excluded CTF fit > 5 Å
12,628 micrographs acceped
Auto blob picking
3.26 million particles picked
2D Classfication (200 classes)
Particles of 48 2D templates
Topaz training
12,628 acceped micrographs
Topaz extracted particles
8.18 million particles picked
1st 3D Reconstruction
Class 1 47.9%
Class 2 35.4%
Class 3 16.7%
15 rounds of heterogenous refinement
2nd 3D Reconstruction
Class 1 48.1%
Class 2 33.8%
Class 3 10.0%
Class 4 8.1%
10 rounds of heterogenous refinement
3rd 3D Reconstruction
Class 1 39.2%
Class 2 32.0%
Class 3 28.8%
5 rounds of heterogenous refinement
Final heterogenous refinement
4.59 Å
4.93 Å
5.78 Å
Class 1 37.8%
Class 2 37.3%
Class 3 24.9%
Re-extract particles, Non-uniform refinement
Non-uniform refinement
Local refinement
Local refinement
Mask up NorA-CDRH3
279,282
3.66 Å
Final map
279,282
3.26 Å

Supplementary Figure 2. Graphical depiction of the cryo-EM data processing workflow. This schematic outlines the key steps involved in the cryo-EM data processing workflow utilized to determine the Coulomb potential map of the NorA sample at pH 5.0 in complex with FabDA1. Solid and dotted boxes are employed to highlight the intact NorA-FabDA1 complex and a variant of the complex in which only the Fv portion of FabDA1 was visible, respectively. Each class is annotated with the number of constituent particles. The percentages indicated in the figure represent the fraction of particles chosen for subsequent refinement stages in relation to the initial total particle count. An identical processing approach was employed for processing cryo-EM micrographs of $NorA^{E222Q,D307N}$, $NorA^{E222Q}$, and $NorA^{D307N}$ in complex with FabDA1.

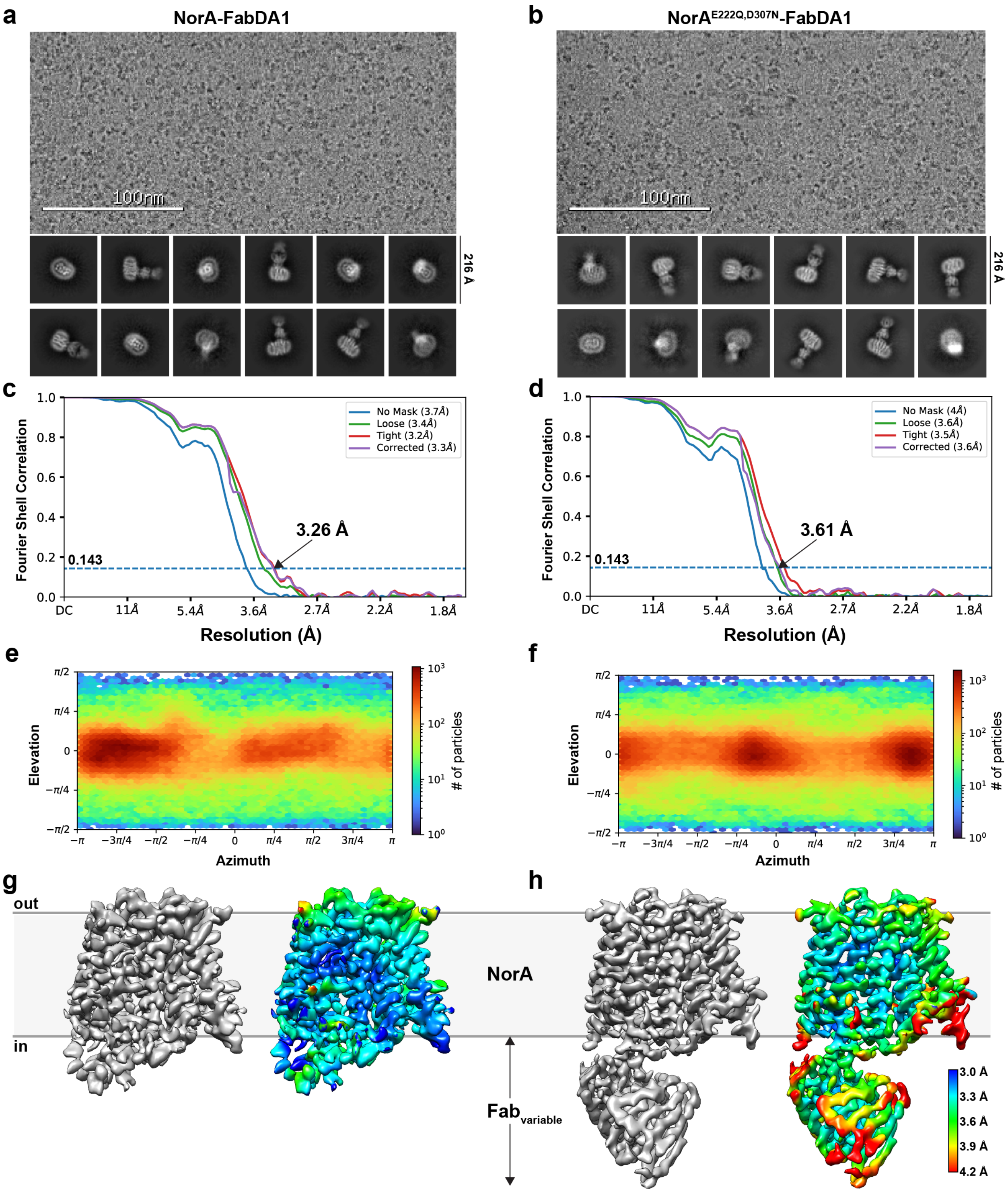

Supplementary Figure 3. Cryo-EM analyses of NorA and NorA^{E222Q,D307N} in complex with FabDA1.

a, b. Representative cryo-EM micrographs (top) and exemplary 2D class averages (bottom) for the NorA-FabDA1 (a) and NorA^{E222Q,D307N}-FabDA1 (b) complexes. The images were obtained using a 300 kV Krios microscope. The most populous 2D classes display intact NorA-Fab complexes in various orientations.

c, d. Fourier shell correlation (top) and directional FSC (bottom) curves were measured for the final NorA (c) and NorA^{E222Q,D307N} (d) reconstructions in complex with FabDA1. An arrow indicates the map resolution at the gold standard FSC value of 0.143.

e, f. Orientation distribution heatmaps depict the final reconstructions for NorA (e) and NorA^{E222Q,D307N} (f) in complex with FabDA1.

g, h. Coulomb potential (left) and local resolution maps (right) are displayed for NorA (g) and NorA^{E222Q,D307N} (h) in complex with FabDA1. The local resolution maps use a consistent linear coloring scale, where blue represents higher resolution regions and red represents lower resolution regions. Note that after masking NorA and NorA^{E222Q,D307N} complexes with FabDA1 displayed cryo-EM density only for the CDRH3 loop and Fv portions of the Fab, respectively.

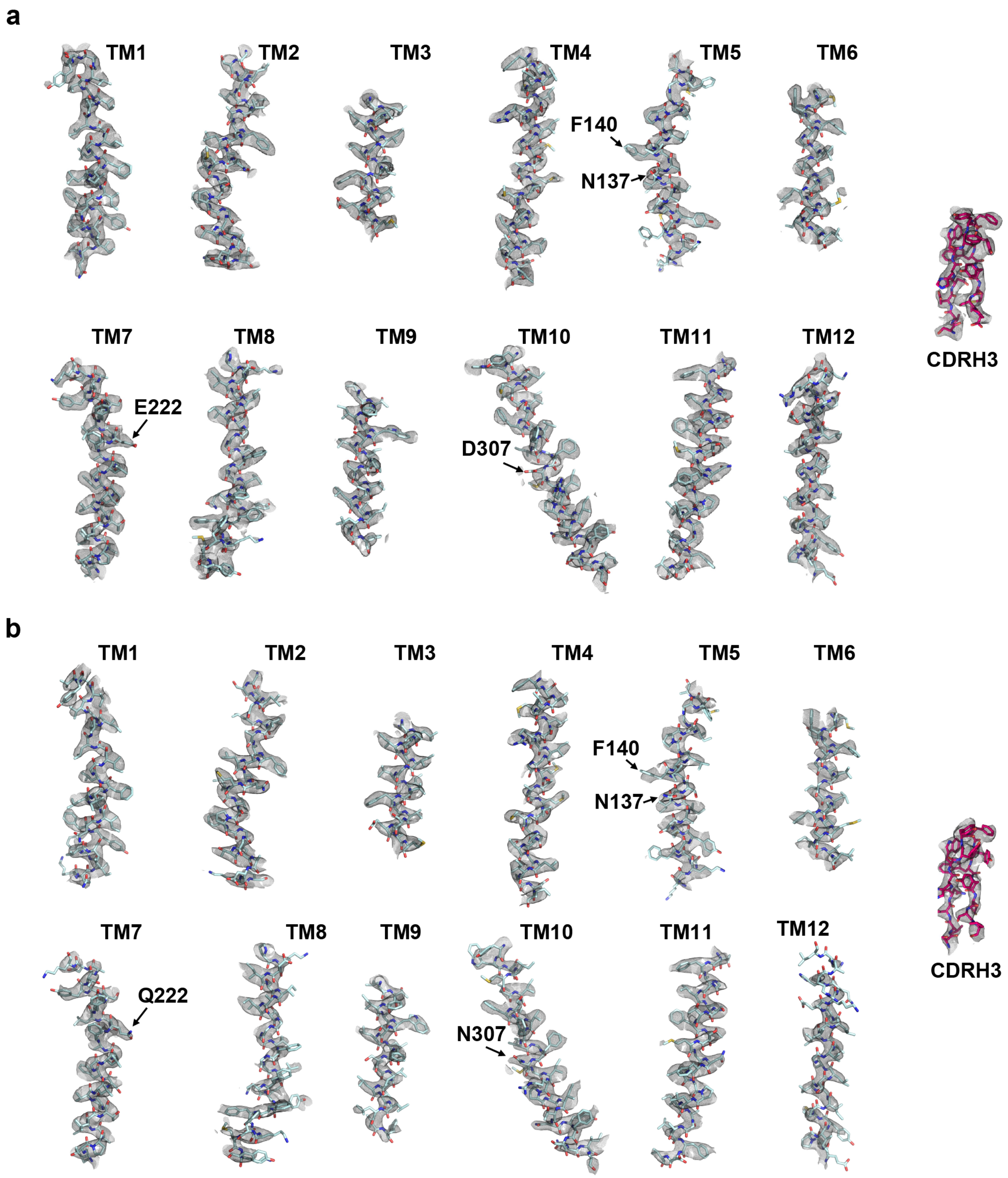

Supplementary Figure 4. Assessment of model-to-map fitting quality of NorA and NorA^{E222Q,D307N} in complex with FabDA1.

a, b. The quality of the NorA (a) and NorA$^{E222Q, D307N}$ (b) structural models in complex with FabDA1 are depicted through their agreement with the respective cryo-EM maps (in a black mesh). The model-to-map fitting is displayed for each transmembrane (TM) helix within NorA and a segment of the CDRH3 loop from FabDA1. The map contour levels were set to 10 sigma units for each TM helix and CDRH3 loop using the *isomesh* command in PyMOL. Each TM domain is defined by the following residues: TM1, 3-29; TM2, 37-65; TM3, 68-84; TM4, 91-119; TM5, 125-151; TM6, 157-176; TM7, 205-232; TM8, 237-265; TM9, 269-286; TM10, 292-321; TM11, 325-353; TM12, 356-382.

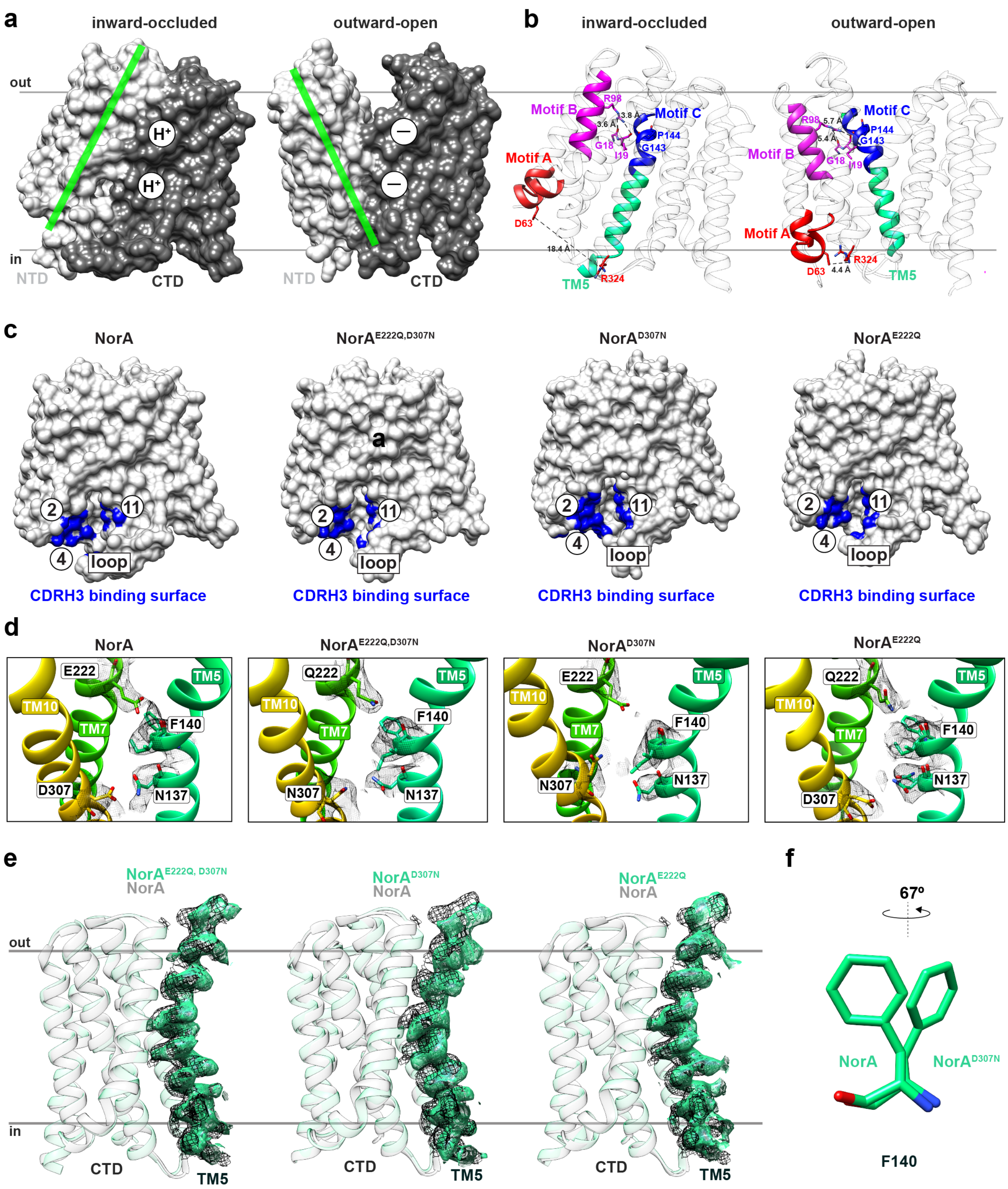

Supplementary Figure 5. Structural views of wild-type NorA and NorA mutants.

a. Surface representations of NorA in the inward-occluded (left) and outward-open (right, PDB ID: 7LO8)[1] conformations. The inward-occluded structure was solved at pH 5.0 while the outward-

open was determined at pH 7.5. The two structures were aligned by residues within the CTD. Green lines estimate the overall orientation of the NTD relative to the two conformations which corresponds to a rotation of ~50°. NTD and CTD were colored in white or grey, respectively.

b. For the inward-occluded structure (wild-type NorA at pH 5.0) and outward-open structure (PDB ID: 7LO8), Motifs A, B, and C are highlighted in red, magenta, and blue, respectively. The distances (in Å) between the carboxyl side chain of Asp63 in Motif A and the backbone amide of Arg324 in the linker between TM10 and TM11, as well as between the side chain amino of Arg98 in Motif B and the backbone carbonyls of Gly18 and Ile19 in TM1, are indicated by black dotted lines. TM5 is colored in spring green.

c. The locations of FabDA1 CDRH3 binding were colored onto the corresponding surface representation of NorA, $\mathrm{NorA}^{\mathrm{E222Q,D307N}}$, $\mathrm{NorA}^{\mathrm{D307N}}$ or $\mathrm{NorA}^{\mathrm{E222Q}}$ in blue. Circled numbers indicate TM helices of NorA that bind to CDRH3 of FabDA1.

d. Assessment of model-to-map fitting quality for Asn137, Phe140, Glu222 (or Gln222), and Asp307 (or Asn307) in NorA, $\mathrm{NorA}^{\mathrm{E222Q,D307N}}$, $\mathrm{NorA}^{\mathrm{D307N}}$ or $\mathrm{NorA}^{\mathrm{E222Q}}$. The contour levels of the cryo-EM maps were set at 0.3 and the zone radii were set at 2.3 Å in Chimera[2].

e. Superimposition of the NorA structure with $\mathrm{NorA}^{\mathrm{E222Q,D307N}}$ (left), $\mathrm{NorA}^{\mathrm{D307N}}$ (middle) or $\mathrm{NorA}^{\mathrm{E222Q}}$ (right). The cryo-EM maps of TM5 were represented in black mesh for NorA and spring green mesh for the mutants. The contour levels of the cryo-EM maps were set at 0.3 and the zone radii were set at 2.0 Å in Chimera[2]. The CTD of NorA was colored in white and the mutants were colored in spring green. Helices were made partially transparent.

f. A comparison of the rotamer of Phe140 between the NorA and $\mathrm{NorA}^{\mathrm{D307N}}$ structures revealed a ~67° change in chi1.

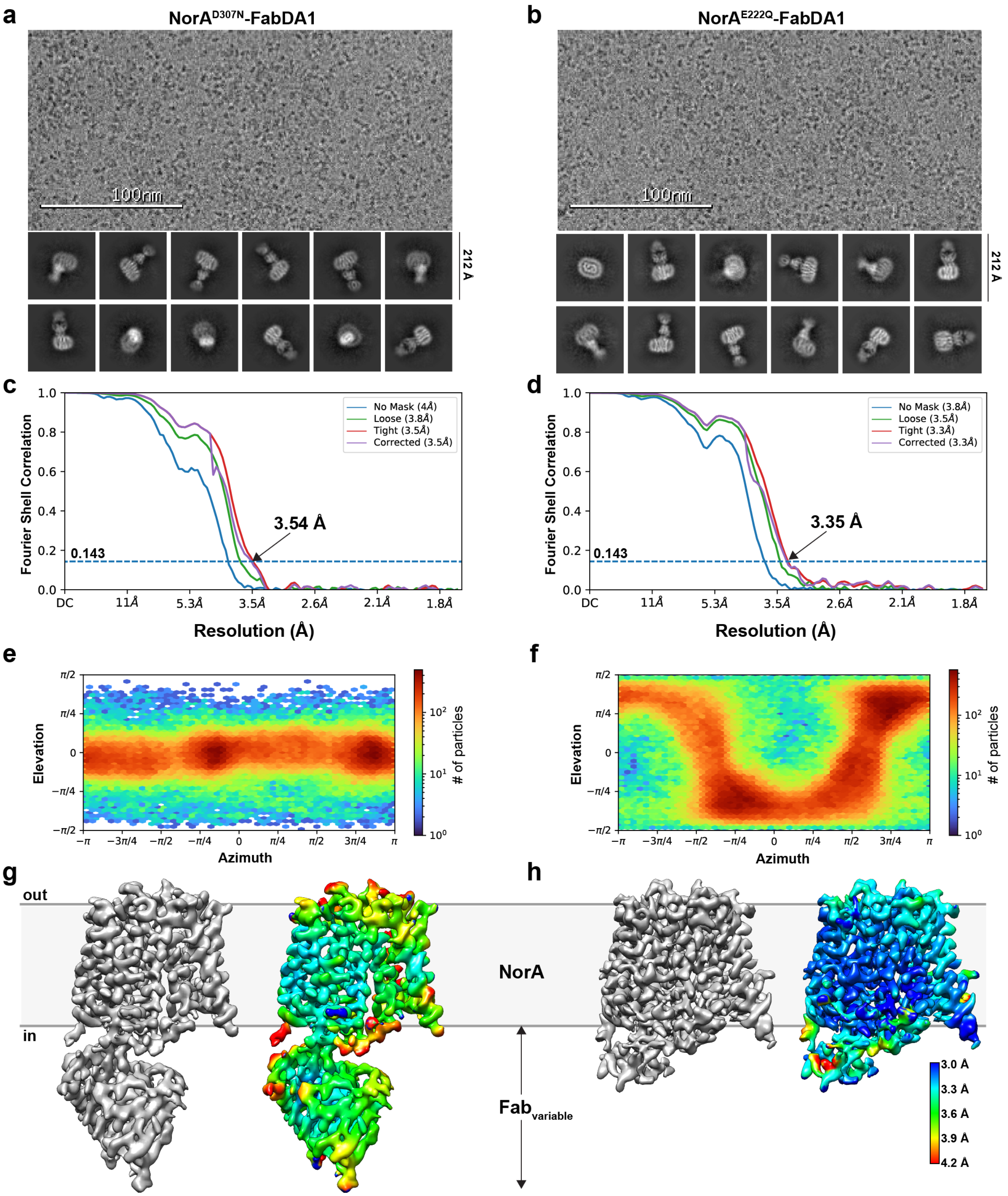

Supplementary Figure 6. Cryo-EM analyses of NorA^{D307N} and NorA^{E222Q} in complex with FabDA1.

a, b. Representative cryo-EM micrographs (top) and exemplary 2D class averages (bottom) illustrating the NorA^{D307N}-FabDA1 (a) and NorA^{E222Q}-FabDA1 (b) complexes. The images were obtained using a 300 kV Krios microscope. The most prevalent 2D classes exhibit intact NorA-Fab complexes in diverse orientations.

c, d. Fourier shell correlation (top) and directional FSC (bottom) curves were computed for the final NorA^{D307N} (c) and NorA^{E222Q} (d) reconstructions in complex with FabDA1. An arrow indicates the map resolution at the gold standard FSC value of 0.143.

e, f. Orientation distribution heatmaps depict the final reconstructions for NorA^{D307N} (e) and NorA^{E222Q} (f) in complex with FabDA1.

g, h. Coulomb potential maps (left) and local resolution maps (right) are presented for NorA^{D307N} (g) and NorA^{E222Q} (h) in complex with FabDA1. These local resolution maps employ a consistent linear color scale, with blue indicating higher resolution regions and red signifying lower resolution regions. Note that after masking NorA^{D307N} and NorA^{E222Q} complexes with FabDA1 displayed cryo-EM density only for the CDRH3 loop and Fv portions of the Fab, respectively.

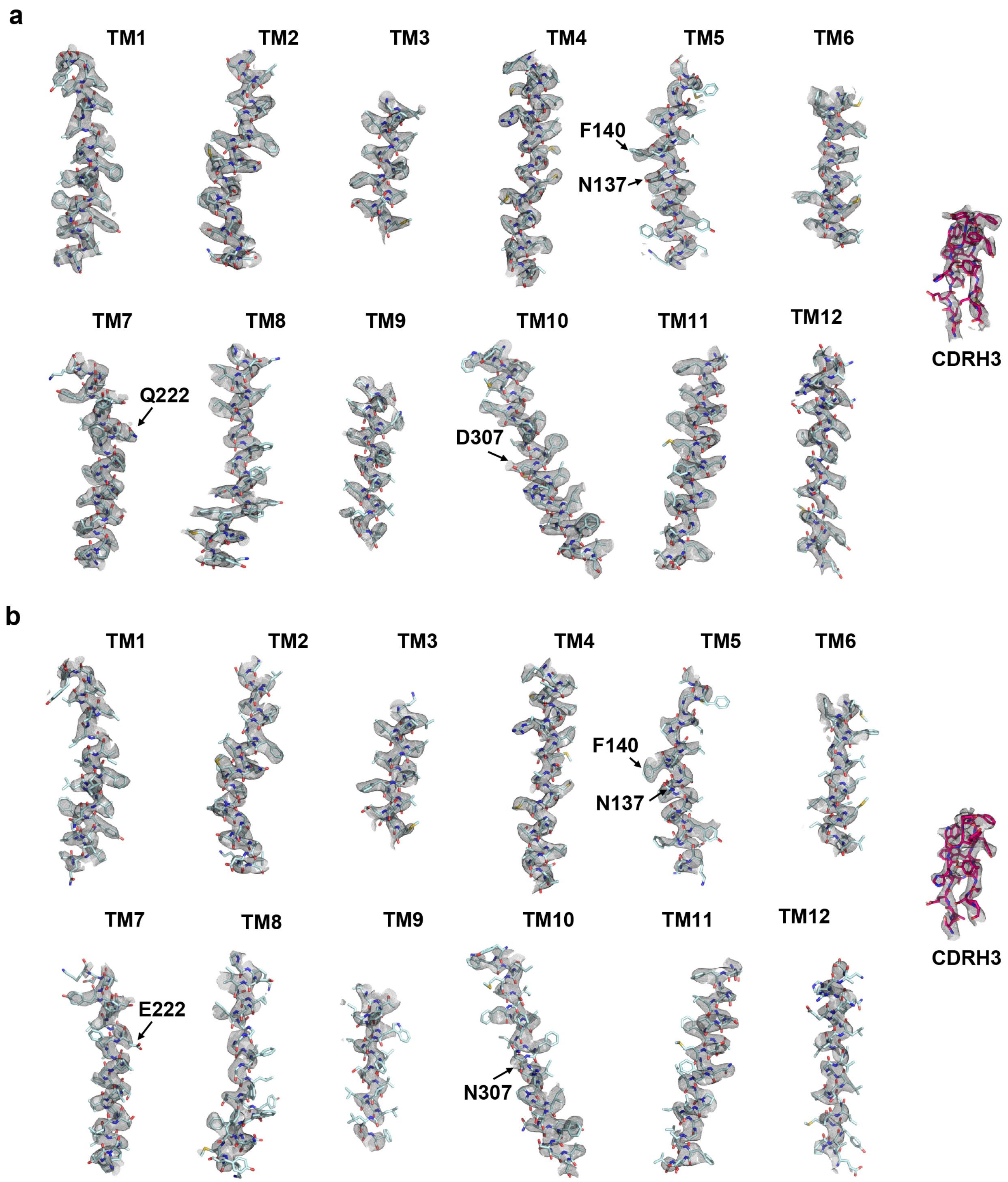

Supplementary Figure 7. Assessment of model-to-map fitting quality of NorA^{E222Q} and NorA^{D307N} in complex with FabDA1.

a, b. The quality of the NorA^{E222Q} (a) and NorA^{D307N} (b) structural models in complex with FabDA1 are depicted through their agreement with the respective cryo-EM maps (in a black mesh). The alignment between the model and map is demonstrated for each TM helix within NorA and a segment of the CDRH3 loop originating from FabDA1. The map contour levels were set to 10 sigma units for each TM helix and CDRH3 loop using the *isomesh* command in PyMOL. Each TM domain is defined by the following residues: TM1, 3-29; TM2, 37-65; TM3, 68-84; TM4, 91-119; TM5, 125-151; TM6, 157-176; TM7, 205-232; TM8, 237-265; TM9, 269-286; TM10, 292-321; TM11, 325-353; TM12, 356-382.

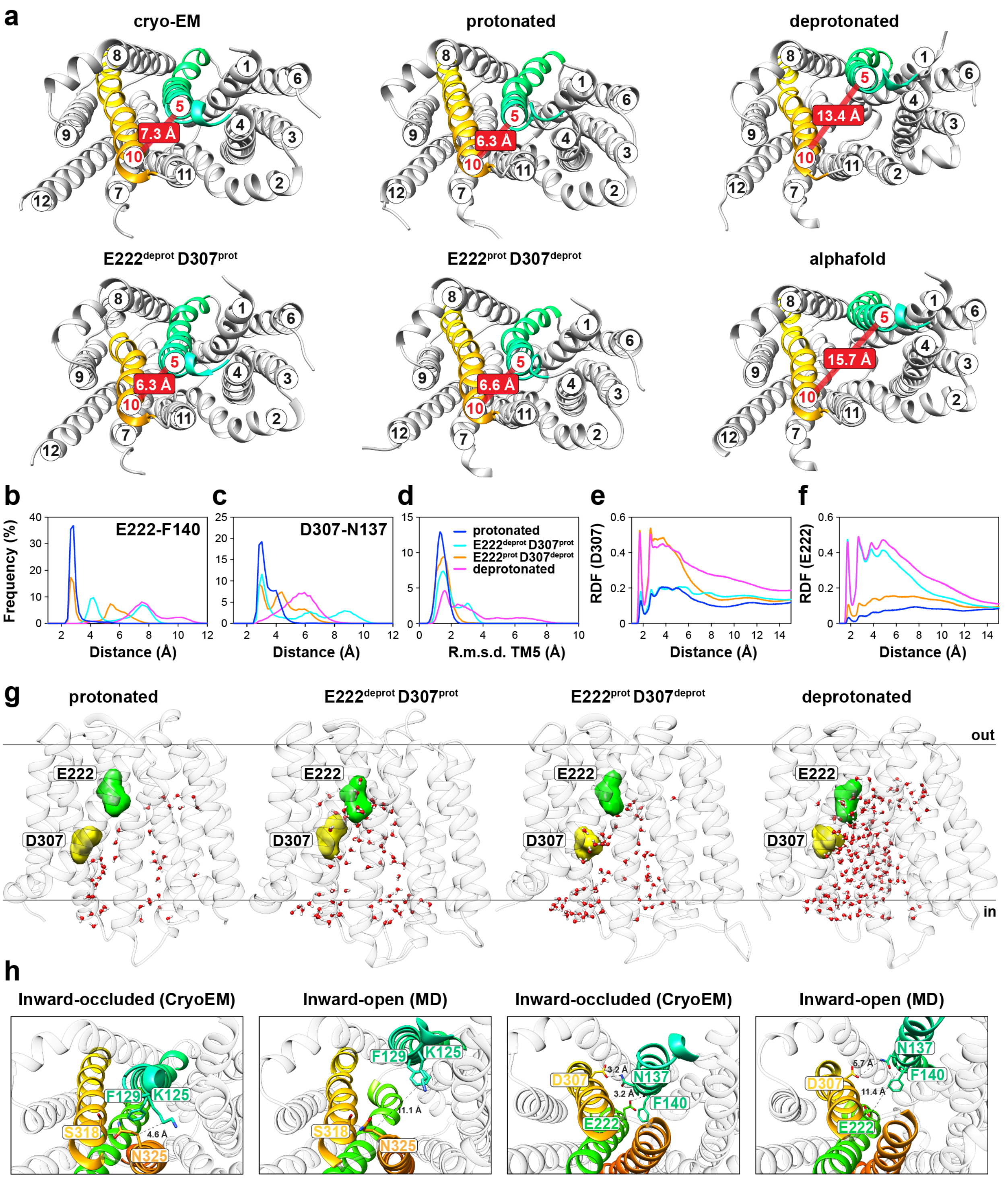

Supplementary Figure 8. Analysis of MD simulations performed on different protonation states of Glu222 and Asp307 within NorA.

a. Cytoplasmic views of the NorA structure determined at pH 5.0 using cryo-EM (upper left), representative MD simulation snapshots of NorA with Glu222/Asp307 protonated (upper middle), Glu222/Asp307 deprotonated (upper right), or singly protonated Asp307 or Glu222 (lower left and lower middle), and the AlphaFold2 predicted inward-open conformation of NorA (lower right). TM helices are numbered and colored as follows: TM5 in spring green, TM10 in gold, and all other helices in grey. The thick red line indicates the distance (in Å) between the Cα atoms of Ala126 in TM5 and Ser318 in TM10.

b, c. Distance distribution for the side chain carboxyl oxygen of Glu222 and the backbone carbonyl oxygen of Phe140 (b) or the side chain carboxyl oxygen of Asp307 and the amide nitrogen/carbonyl oxygen of Asn137 (c) for NorA MD simulations where Glu222 and Asp307 were protonated (blue), Glu222 or Asp307 was protonated (cyan for $E222^{deprot}D307^{prot}$; orange for $E222^{prot}D307^{deprot}$), or Glu222 and Asp307 were deprotonated (magenta).

d. Backbone r.m.s.d. from MD simulations for TM5 of NorA plotted as a frequency distribution where Glu222 and Asp307 were protonated (blue), Glu222 or Asp307 was protonated (cyan for $E222^{deprot}D307^{prot}$; orange for $E222^{prot}D307^{deprot}$), or Glu222 and Asp307 were deprotonated (magenta). The r.m.s.d. was calculated for residues Lys125 to Leu142 in TM5 after aligning the structures to the CTD.

e, f. The water radial distribution function (RDF) surrounding Asp307 (e) and Glu222 (f) calculated from NorA MD simulations where Glu222 and Asp307 were protonated (blue), Glu222 or Asp307 was protonated (cyan for $E222^{deprot}D307^{prot}$; orange for $E222^{prot}D307^{deprot}$), or Glu222 and Asp307 were deprotonated (magenta).

g. Representative snapshots from MD simulations of the water distribution in the substrate binding pocket of NorA protonated at Glu222 and Asp307 (left), protonated at Glu222 or Asp307 (middle),

and deprotonated at Glu222 and Asp307 (right). Water molecules are depicted as red spheres for oxygens and white spheres for hydrogens, Glu222 and Asp307 are highlighted in spring green and yellow surfaces, respectively, and TM helices are shown in light grey.

h. Structural views of the inward-open conformation of wild-type NorA determined by cryo-EM and a snapshot of the inward-open conformation determined using MD simulations. The left two panels reveal potential gating residues in the inward-occluded to inward-open transition, including Lys125, Phe129, Ser318, and Asn325. The right two panels display differences for Glu222, Asp307, and surrounding residues between the two conformations. TM helices are colored as follows: TM5 in spring green, TM7 in green, TM10 in gold, TM11 in orange, and all other helices in grey. Dashed black lines correspond to the indicated distances (in Å).

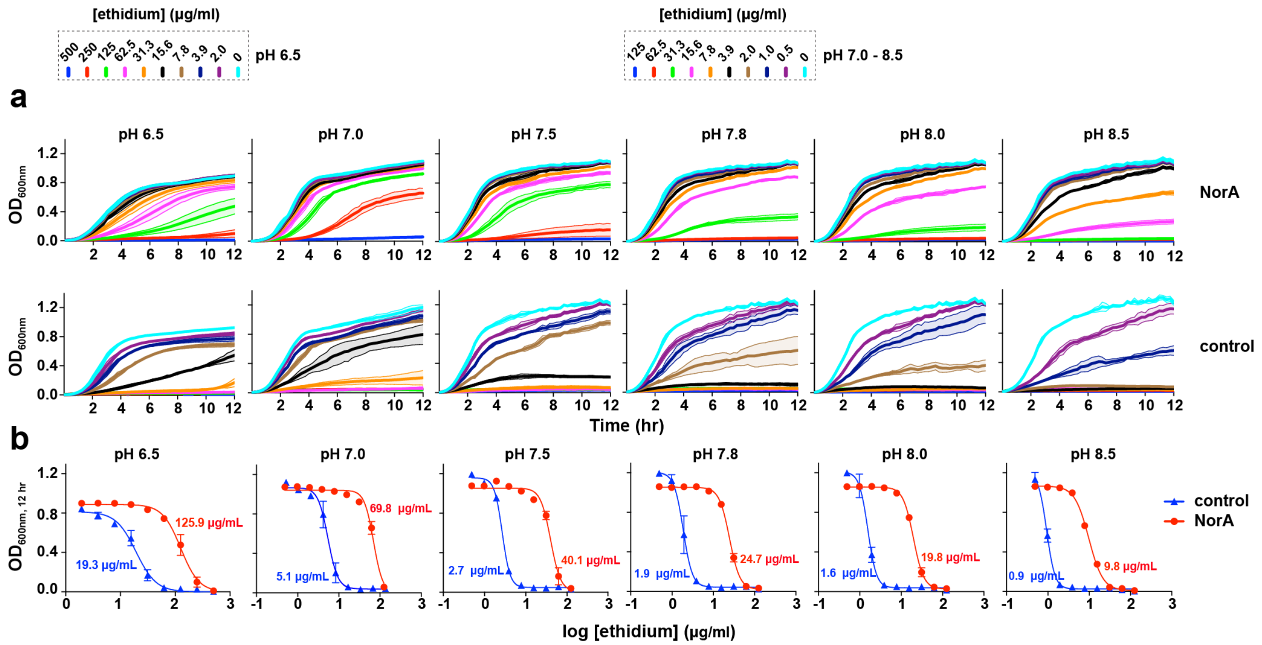

Supplementary Figure 9. Growth inhibition assay of NorA in MRSA at different external pH values.

a. Growth inhibition experiments of MRSA$^{\Delta norA}$ transformed with a hemin-inducible plasmid containing the wild-type *norA* gene (top row) or a dead *norA* mutant control (bottom row) in the presence of the indicated pH values of the growth medium and the ethidium bromide concentrations in the legend. Expression of the *norA* gene was induced with 1 µM hemin. Solid lines represent the average values, while the shaded regions in the same color indicates the standard deviation, derived from four technical replicates in a single representative experiment. Consistent findings were confirmed in a minimum of two independent experiments.

b. Growth inhibition experiments of MRSA$^{\Delta norA}$ transformed with a hemin-inducible plasmid containing the wild-type *norA* gene (red circles) or a dead *norA* mutant control (blue triangles) in the presence of variable ethidium bromide concentration (0 to 125 µg/ml or 0 to 500 µg/ml). Growth is displayed by the OD_{600nm} value at the 12 hr timepoint. Solid lines correspond to non-linear fits of experimental data to obtain IC_{50} values. The error ranges at the 95% confidence

interval associated with the IC_{50} values of ethidium bromide for NorA were: 117.6 -134.2 μg/mL at pH 6.5, 66.1 - 72.8 μg/mL at pH 7.0, 37.4 - 42.9 μg/mL at pH 7.5, 24.0 - 25.3 μg/mL at pH 7.8, 19.2 - 20.2 μg/mL at pH 8.0, and 9.4 - 10.1 μg/mL at pH 8.5. The error ranges at the 95% confidence interval associated with the IC_{50} values of ethidium bromide in the control samples were: 14.1 - 20.7 μg/mL at pH 6.5, 4.5 - 5.4 μg/mL at pH 7.0, 2.5 - 2.8 μg/mL at pH 7.5, 1.8 - 2.0 μg/mL at pH 7.8, 1.5 - 1.6 μg/mL at pH 8.0, and 0.9 - 1.0 μg/mL at pH 8.5.

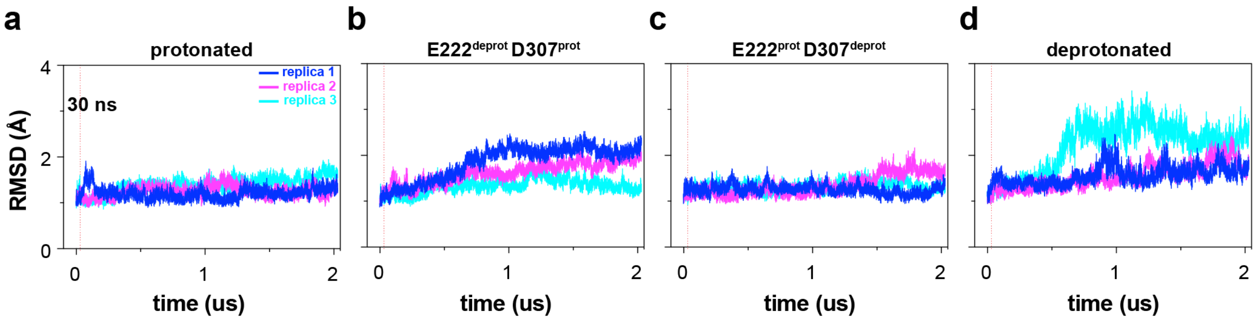

Supplementary Figure 10. Backbone r.m.s.d. of MD simulations.

a-d. Backbone r.m.s.d. of MD simulations initiated from the structure of the proton-bound NorA (8TTE [https://doi.org/10.2210/pdb8TTE/pdb]). MD simulations were conducted on NorA in the following protonation states: Glu222/Asp307 protonated (**a**), Glu222 deprotonated and Asp307 protonated (**b**), Glu222 protonated and Asp307 deprotonated (**c**), and Glu222/Asp307 deprotonated (**d**). Each state of NorA was simulated with three replicates, represented by different colors. Analyses in subsequent panels were conducted after 30 nsec, as indicated by the red dotted lines. Residues from Met1 to Ile177 and Val203 to Lys382 were used to calculate the backbone r.m.s.d. since these were the ordered parts of NorA observed by cryo-EM.

Supplementary Table 1. Cryo-EM data collection and structure determination of NorA and mutants of NorA in complex with FabDA1.

	NorA	NorA$^{E222Q, D307N}$	NorA^{D307N}	NorA^{E222Q}
**EMDB ID**	EMD-41605	EMD-41606	EMD-41608	EMD-41607
**PDB ID**	8TTE	8TTF	8TTH	8TTG
**Data Collection**				
Magnification (x)	105,000	105,000	105,000	105,000
Voltage (kV)	300	300	300	300
Number of movies	12,953	11,811	10,461	8,884
Electron dose ($e^-/Å^2$)	50.25	50.25	52.96	52.96
Defocus range (µm)	-1.0 – -1.8	-1.0 – -1.8	-1.0 – -2.0	-1.0 – -2.0
Collection mode	Super-resolution	Super-resolution	Super-resolution	Super-resolution
Effective pixel size (Å)	0.422	0.422	0.4124	0.4124
**Data processing**				
Initial number of particles	8,177,406	9,697,576	6,901,412	6,707,069
Final number of particles	279,282	406,620	136,284	246,742
Symmetry imposed	C1	C1	C1	C1
B - factor	163.6	183.7	178.5	179.2
Map resolution* (Å)	3.26	3.61	3.54	3.35
**Model refinement**				
Non-hydrogen atoms	2,992	4,764	4,621	3027
Protein residues	385	621	603	389
Mean B factor				
Protein ($Å^2$)	63.57	64.5	66.73	50.41
RMS deviations				
Bond lengths (Å)	0.006	0.003	0.003	0.004
Bond angles (°)	0.915	0.598	0.686	0.743
MolProbity score	1.89	1.58	1.76	1.67
Clash score	10.88	9.19	12.40	12.05
Rotamer outliers (%)	0.94	0.00	0.20	0.62
Ramachandran plot				
Favored (%)	95.78	97.56	97.14	97.65
Allowed (%)	4.22	2.44	2.86	2.35
Outliers (%)	0.00	0.00	0.00	0.00
Model resolution† (Å)	3.6	3.9	3.9	3.7

* Resolution determined by Fourier shell coefficient threshold of 0.143 for corrected masked map.
† Resolution determined between the model and the sharpened map by Fourier shell coefficient threshold of 0.5.

Supplementary Table 2: MD simulation setup system details

	NorA E222^{prot} D307^{prot}	NorA E222^{deprot} D307^{deprot}	NorA E222^{deprot} D307^{prot}	NorA E222^{prot} D307^{deprot}
**Starting PDB**	8TTE	8TTE	8TTE	8TTE
**Simulation box dimensions (Å)**	81x 70 x 105	81 x 70 x 105	81 x 70 x 105	81 x 70 x 105
**Total atoms**	54,361	54,379	54,490	54,288
**Water molecules**	10,749	10,755	10,792	10,722
**NaCl concentration**	100 mM	100 mM	100 mM	100 mM
**Number of DOPE lipids**	93	93	93	93
**Number of DOPG lipids**	31	31	31	31

References

1 Brawley, D. N. *et al.* Structural basis for inhibition of the drug efflux pump NorA from Staphylococcus aureus. *Nat Chem Biol* **18**, 706-712 (2022). https://doi.org/10.1038/s41589-022-00994-9

2 Pettersen, E. F. *et al.* UCSF Chimera--a visualization system for exploratory research and analysis. *J Comput Chem* **25**, 1605-1612 (2004). https://doi.org/10.1002/jcc.20084
