# Supplementary material for: Proton-coupled transport mechanism of the efflux pump NorA
Source: Nat Commun. 2024 May 27;15:4494. doi: 10.1038/s41467-024-48759-3 (PMC11130294; doi:10.1038/s41467-024-48759-3)
Supplement: Supplementary file 3 — Reporting Summary [file 41467_2024_48759_MOESM3_ESM.pdf]

Corresponding author(s): Da-Neng Wang
Nathaniel J. Traaseth

Last updated by author(s): May 2, 2024

Reporting Summary

Nature Research wishes to improve the reproducibility of the work that we publish. This form provides structure for consistency and transparency in reporting. For further information on Nature Research policies, see our Editorial Policies and the Editorial Policy Checklist.

Statistics

For all statistical analyses, confirm that the following items are present in the figure legend, table legend, main text, or Methods section.

n/a	Confirmed	
☐	☒	The exact sample size (*n*) for each experimental group/condition, given as a discrete number and unit of measurement
☐	☒	A statement on whether measurements were taken from distinct samples or whether the same sample was measured repeatedly
☐	☒	The statistical test(s) used AND whether they are one- or two-sided<br>*Only common tests should be described solely by name; describe more complex techniques in the Methods section.*
☒	☐	A description of all covariates tested
☒	☐	A description of any assumptions or corrections, such as tests of normality and adjustment for multiple comparisons
☐	☒	A full description of the statistical parameters including central tendency (e.g. means) or other basic estimates (e.g. regression coefficient) AND variation (e.g. standard deviation) or associated estimates of uncertainty (e.g. confidence intervals)
☐	☒	For null hypothesis testing, the test statistic (e.g. *F*, *t*, *r*) with confidence intervals, effect sizes, degrees of freedom and *P* value noted<br>*Give P values as exact values whenever suitable.*
☒	☐	For Bayesian analysis, information on the choice of priors and Markov chain Monte Carlo settings
☒	☐	For hierarchical and complex designs, identification of the appropriate level for tests and full reporting of outcomes
☒	☐	Estimates of effect sizes (e.g. Cohen's *d*, Pearson's *r*), indicating how they were calculated

Our web collection on statistics for biologists contains articles on many of the points above.

Software and code

Policy information about availability of computer code

Data collection	Cryo-EM data: Legionon 3.6(44)<br>Growth inhibition: Bioscreen C Pro<br>MST binding assay: MoControl v1.6.1<br>Ethidium bromide efflux assay: SoftMax Pro 7.1.2<br>MD simulations: GROMACS 2020.4
Data analysis	Cryo-EM data processing: cryoSPARC v.3.3.1<br>Construction of structural models: Namdinator, Coot 0.9.8.92 EL (ccp4) and Phenix-1.20.1-4487<br>Map and model viewing: Chimera-1.15 or ChimeraX-1.7<br>Growth inhibition, MST binding assay and efflux assy: Prism software version 9<br>MD simulations: GROMACS 2020.4, VMD 1.9.3 and Prism software version 9

For manuscripts utilizing custom algorithms or software that are central to the research but not yet described in published literature, software must be made available to editors and reviewers. We strongly encourage code deposition in a community repository (e.g. GitHub). See the Nature Research guidelines for submitting code & software for further information.

Data

Policy information about availability of data

All manuscripts must include a data availability statement. This statement should provide the following information, where applicable:

- Accession codes, unique identifiers, or web links for publicly available datasets
- A list of figures that have associated raw data
- A description of any restrictions on data availability

The datasets generated during and/or analyzed during the current study are deposited. The cryo-EM maps have been deposited in the Electron Microscopy Data Bank (EMDB) under accession codes EMD-41605 [https://www.ebi.ac.uk/pdbe/entry/emdb/EMD-41605] for the EMDB entry of NorA; EMD-41606 [https://www.ebi.ac.uk/pdbe/entry/emdb/EMD-41606] for the EMDB entry of NorAE222Q,D307N; EMD-41608 [https://www.ebi.ac.uk/pdbe/entry/emdb/EMD-41608] for the EMDB entry of NorAD307N; (EMD-41607 [https://www.ebi.ac.uk/pdbe/entry/emdb/EMD-41607] for the EMDB entry of NorAE222Q. The atomic coordinates have been deposited in the Protein Data Bank (PDB) under accession codes 8TTE [https://doi.org/10.2210/pdb8TTE/pdb] for the PDB entry of NorA; 8TTF [https://doi.org/10.2210/pdb8TTF/pdb] for the PDB entry of NorAE222Q,D307N; 8TTH [https://doi.org/10.2210/pdb8TTH/pdb] for the PDB entry of NorAD307N; 8TTG [https://doi.org/10.2210/pdb8TTG/pdb] for the PDB entry of NorAE222Q. The published NorA PDB codes of 7LO7 [https://doi.org/10.2210/pdb7LO7/pdb] and 7LO8 [https://doi.org/10.2210/pdb7LO8/pdb] were referred to in this work. The input for MD simulations and the coordinates of starting and ending structures are accessible via the following link: https://doi.org/10.5281/zenodo.11075313.

Supplementary Information is available for this paper. The source data underlying Figures 3b, 5b-c, 6b-e, and Supplementary Figure 1, 8b-f, 9 are provided as a Source Data file. Correspondence and requests for materials should be addressed to traaseth@nyu.edu or da-neng.wang@med.nyu.edu.

Field-specific reporting

Please select the one below that is the best fit for your research. If you are not sure, read the appropriate sections before making your selection.

☒ Life sciences ☐ Behavioural & social sciences ☐ Ecological, evolutionary & environmental sciences

For a reference copy of the document with all sections, see nature.com/documents/nr-reporting-summary-flat.pdf

Life sciences study design

All studies must disclose on these points even when the disclosure is negative.

Sample size	Binding and growth inhibition assays were performed in duplicate (or more) to ensure reproducibility based on prior experimental experience and our previous publication on NorA (Brawley et al. 2022. Structural basis for inhibition of the drug efflux pump NorA from Staphylococcus aureus. Nat Chem Biol 18, 706-712. https://doi.org/10.1038/s41589-022-00994-9). The sample sizes are listed for each sample in the Methods section and/or in the figure legends.
Data exclusions	No data were excluded from the analyses.
Replication	Multiple experimental datasets were used to confirm experimental results. Attempts to replicate the central findings of the manuscript were successful. Independent experiments were performed to confirm the reproducibility.
Randomization	Randomization was not relevant in our study; the people who performed experiments needed to analyze datasets.
Blinding	Blinding was not relevant in our study; the people who performed experiments needed to analyze datasets.

Reporting for specific materials, systems and methods

We require information from authors about some types of materials, experimental systems and methods used in many studies. Here, indicate whether each material, system or method listed is relevant to your study. If you are not sure if a list item applies to your research, read the appropriate section before selecting a response.

Materials & experimental systems

n/a	Involved in the study	
☐	☒	Antibodies
☒	☐	Eukaryotic cell lines
☒	☐	Palaeontology and archaeology
☒	☐	Animals and other organisms
☒	☐	Human research participants
☒	☐	Clinical data
☒	☐	Dual use research of concern

Methods

n/a	Involved in the study	
☒	☐	ChIP-seq
☒	☐	Flow cytometry
☒	☐	MRI-based neuroimaging

Antibodies

Antibodies used	1. c-Myc tag antibody, Genscript, catalogue number: A00704, lot number: SLBW4917<br>2. Anti-Mouse IgG, Sigma, catalogue number: A4416, lot number: 19G001868<br>3. SrtA antibody: gifted from other lab.
Validation	Each primary antibody (mouse) was validated using immunoblot analyses. Namely, negative and positive controls were analyzed with the primary antibodies using samples containing the absence and presence of tagged proteins, respectively.
